# Changes in Oxidative Stress and Antioxidant Enzyme Activities in Streptozotocin-Induced Diabetes Mellitus in Rats: Role of* Alhagi maurorum* Extracts

**DOI:** 10.1155/2016/5264064

**Published:** 2016-01-18

**Authors:** S. A. Sheweita, S. Mashaly, A. A. Newairy, H. M. Abdou, S. M. Eweda

**Affiliations:** ^1^Department of Biotechnology, Institute of Graduate Studies and Research, Alexandria University, Egypt; ^2^Department of Biochemistry, Faculty of Science, Alexandria University, Egypt; ^3^Department of Zoology, Faculty of Science, Alexandria University, Egypt

## Abstract

*Alhagi maurorum* (camel thorn plant) is a promising medicinal plant due to the presence of flavonoids and phenolic compounds as major contents of its constituents. No previous study has been conducted before on* A. maurorum extracts* as an antioxidative stress and/or antidiabetic herb in STZ-induced DM in rats. Therefore, four groups of rats were allocated as control (C), STZ-induced DM (D), and STZ-induced DM supplemented with 300 mg/kg BW of either aqueous extract (WE) or ethanolic extract (EE) of* A. maurorum*. The plasma levels of glucose, TG, TC, LDL-C and VLDL-C, MDA, and bilirubin and the activities of transaminases and GR were significantly increased in the diabetic group. Also, diabetic rats showed severe glucose intolerance and histopathological changes in their livers. In addition, levels of insulin, total proteins, GSH, and HDL-C and the activities of SOD, GPx, and GST were significantly decreased in the diabetic rats compared to those of the control group. The ingestion of* A. maurorum* extracts lowered the blood glucose levels during the OGTT compared to the diabetic rats and restored all tested parameters to their normal levels with the exception of insulin level that could not be restored. It is concluded that* A. maurorum* extracts decreased elevated blood glucose levels and hyperlipidemia and suppressed oxidative stress caused by diabetes mellitus in rats.

## 1. Introduction

The incidence of diabetes mellitus has been increased annually all over the world and the number of diabetic patients will jump from 382 million patients in year 2013 to 592 million in year 2035 [1]. The majority of diabetic patients are non-insulin-dependent and relatively small proportions (7–10%) of diabetic patients have insulin-dependent diabetes (T1D) [2]. Type 1 diabetes (T1D) is a chronic disease that results from an autoimmune destruction of *β*-cells of the pancreas. Therefore, insulin deficiency and hyperglycemia are the main outcomes of T1D [3]. This may generate an array of disturbances in glucose and lipid homeostasis resulting in hyperglycemia and dyslipidemia [4]. Persistent hyperglycemia in diabetes causes increased production of oxygen free radicals from autoxidation of glucose [5] and glycosylation of protein [6] which lead to oxidative stress which is associated with several health complications including antipathies, cardiovascular disorders, blindness, renal failure, neuropathies, and cancers [3, 7].

Recently, drug formulation from natural herbs, for treatment of diabetes mellitus drugs and other diseases, attracted the attention of many researchers [8].* Alhagi maurorum* (Leguminosae) also called camel thorn plant or aqool is a favorable food for camels. It is widely distributed in Asia, the Middle East, Europe, and Africa [9]. It has been used as diaphoretic, diuretic, expectorant, and ulcer treatment [10]. Oil from its leaves was used for rheumatoid treatment and as laxative [11, 12]. Water extract of the roots is used to enlarge the ureter and to remove the kidney stones, whereas the methanolic extract is used as an antidiarrheal agent [13] and as herbal cough syrup [12].* A. maurorum* species contains fatty acids and sterols, flavonoids, coumarins, alkaloids, and vitamins. In addition, six main flavonoid glycosides were isolated from the ethanolic extract of* A. maurorum* [10]. Moreover,* A. maurorum* roots contain lupeol [11], which is used as an antiangiogenic, antioxidative, and anti-inflammatory agent [14, 15].

Streptozotocin-treated rats developed clinical features and signs, which are similar to those found in type 1 diabetes mellitus [16]. To the best of our knowledge, no previous studies have been conducted before to investigate the antidiabetic effects of ethanolic and aqueous extracts of* A. maurorum* through determination of blood glucose level, free radicals, antioxidant enzymes, and lipid profile in STZ-induced diabetic rats. Therefore, the present study was undertaken to investigate the effectiveness of* A. maurorum* extracts in STZ-induced diabetic rats and to evaluate their therapeutic potential for treatment of diabetes mellitus.

## 2. Methods and Materials

### 2.1. Preparation of Ethanolic and Aqueous Extracts of* Alhagi maurorum*

Camel thorn plant (*A. maurorum*) was collected from Wadi El Natrun region (Egypt) after getting the agreement of the Director of Wilderness Areas in El-Beheira Governorate and authentication by Salama El Darer, Professor of Plant Ecology, Botany and Microbiology Department, Faculty of Science, Alexandria University. We confirm that no specific permission was required for collection this plant from Wadi El Natrun region because it is a desert. Moreover, we confirm that this field study did not cause any danger for any plant in this area. The aerial parts of* A. maurorum* were collected, washed three times with tap water and two times with distilled water, dried in the shade, and milled to fine powder by Wiley mill (Model 4-GMI, Germany). Ground plant (100 g) was refluxed with 1 liter of 70% ethanol or with 1 liter of double distilled water for 1 hour. After filtration, solvents were removed under reduced pressure at 40°C using rotary evaporator (Buchi, Model 462, Germany) and freeze dried by lyophilizer to obtain the dried extracts. Aqueous suspensions were prepared from ethanolic or aqueous extracts and administered to rats orally.

### 2.2. Determination of Total Phenolic and Flavonoid Contents of* Alhagi maurorum* Extracts

Total phenolic contents of ethanolic and aqueous extracts of* A. maurorum* were determined by the Folin-Ciocalteu method [17]. Gallic acid (0–0.9 mg/dL) was used as standard for phenolic compounds. The data were expressed as milligram gallic acid equivalents/g lyophilized powder. The total flavonoid content was determined using aluminum chloride colorimetric method as described earlier [18]. Rutin (0–20 mg/L) was used as a standard for flavonoid. The results were expressed as milligram rutin equivalents/g lyophilized powder.

### 2.3. Induction of Diabetes Mellitus by Streptozotocin

Type 1 diabetes mellitus was induced in rats by the intraperitoneal injection of freshly prepared streptozotocin (STZ) at a dose of 45 mg/kg dissolved in 0.1 M citrate buffer solution BW [19]. Three days after the STZ injection, the blood was withdrawn from the tail vein, and the glucose level was determined. Rats were diabetic when their fasting blood glucose levels were more than 200 mg/dL.

### 2.4. Experimental Design

Forty healthy, mature male Albino rats were provided by the Animal House of the Faculty of Medicine, Alexandria University, Egypt. The average weight of rats was 140 g and maintained in wire-bottomed cages. Rats had free access to food and water and were kept at 25 ± 2°C and 50–60% humidity. The protocol of animals handling was approved by the ethical guidelines prescribed by the MRI, Alexandria University, Alexandria, Egypt. After induction of diabetes, the diabetic rats were randomly allocated to 3 groups (10 rats each): (1) diabetic group (D-group); (2) diabetic group receiving 300 mg/kg BW water extract of* A. maurorum* (WE-group); and (3) diabetic group receiving 300 mg/kg BW ethanolic extract of* A. maurorum* (EE-group). Ten rats received distilled water and were used as control group (C-group). The* A. maurorum* extracts were suspended in distilled water and administered orally as a daily dose for four weeks. Rats were fasted overnight and euthanized by cervical dislocation. Blood samples were collected in EDTA-coated tubes. Livers and pancreases were removed, washed with cold saline, and stored at −80°C.

### 2.5. Oral Glucose Tolerance Test and Assay of Biochemical Parameters

Briefly, after overnight fasting, rats were intragastrically loaded with glucose (2 g/kg). Blood samples were withdrawn from the tail vein at 0, 30, 60, 90, and 120 minutes and the blood glucose levels were determined using commercial kit as described by Tietz [20]. Nonradioactive assay was used for determination of insulin level in plasma of rats according to the manufacturer's protocol [21]. Triglyceride, total cholesterol, LDL-C and HDL-C, aspartate aminotransferase (AST), alanine aminotransferase (ALT), and total bilirubin were measured in the plasma using commercial kits from Bio-System Company (Egypt). The protein content was determined according to the method of Lowry et al. [22].

### 2.6. Evaluation of Oxidative Stress Markers

Lipid peroxidation was evaluated by measuring thiobarbituric acid reactive substances (TBARS) according to the method of Draper and Hadley [23]. Reduced glutathione was measured spectrophotometrically as described by Shaikh et al. [24]. Liver superoxide dismutase was estimated according to the method of S. Marklund and G. Marklund [25]. Glutathione peroxidase activity in the liver supernatant was measured according to the method of Flohe and Gunzler [26]. The hepatic activity of glutathione reductase was assayed according to the method of Smith et al. [27]. The glutathione-S-transferase activity was measured according to the method of Habig et al. [28].

### 2.7. Histological Analysis

Specimens of pancreas and liver tissues of the different groups were immediately fixed in 10% formalin and then treated with conventional grade of alcohol and xylene. For histopathological examination, 6 *μ*M specimens thicknesses of both pancreas and livers were stained with hematoxylin and eosin (H&E) stains[29].

### 2.8. Statistical Analyses

Statistical analysis was performed using SPSS software package (Version 17.0). The data were analyzed using one-way analysis of variance (ANOVA) and the differences between means of all groups were tested using Least Significant Difference (LSD). Probability value less than 0.05 was considered statistically significant.

## 3. Results

### 3.1. The Total Flavonoids and Phenolic Contents of* Alhagi* Extracts

The results of the present study showed that concentrations of the total phenolic and flavonoid compounds in the aqueous extract of* A. maurorum* were gallic acid (90.87 ± 1.5 mg) and rutin (5.20 ± 0.24 mg) equivalent/100 g dried-weight, respectively, while the contents of the ethanolic extract of* A. maurorum* from these components were gallic acid (105.16 ± 2.6 mg) and rutin (6.16 ± 0.27 mg) equivalent/100 g dried-weight, respectively.

### 3.2. *Alhagi* Extracts and Biochemical Parameters

**Figure 1:**
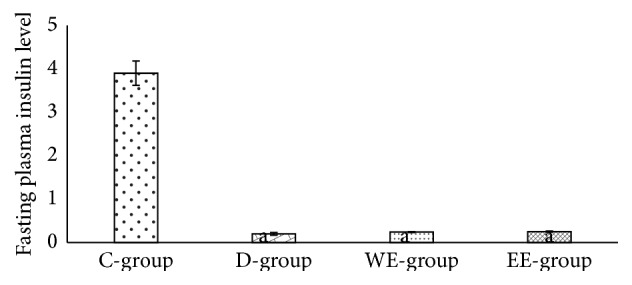
The fasting insulin level in diabetic rats treated with 300 mg/kg BW of either water or ethanolic extract of* Alhagi maurorum*.

Activities of ALT and AST and the level of the total bilirubin were significantly increased (*P* ≤ 0.05) in diabetic rats compared to those of control group. Water and ethanolic extracts of* A. maurorum* exhibited significantly improved hepatic function of the diabetic rats (Table 1). The fasting blood glucose levels of diabetic rats were significantly (*P* ≤ 0.05) higher than those of the control group. However, administration of water and/or ethanolic extract of* A. maurorum* to the diabetic group significantly (*P* ≤ 0.001) restored their fasting blood glucose levels to the control value. Plasma insulin levels were significantly (*P* ≤ 0.05) decreased in the diabetic group compared to the control group. However, WE and EE did not restore the plasma insulin levels in diabetic groups to the normal level (Figure 1).

**Figure 2:**
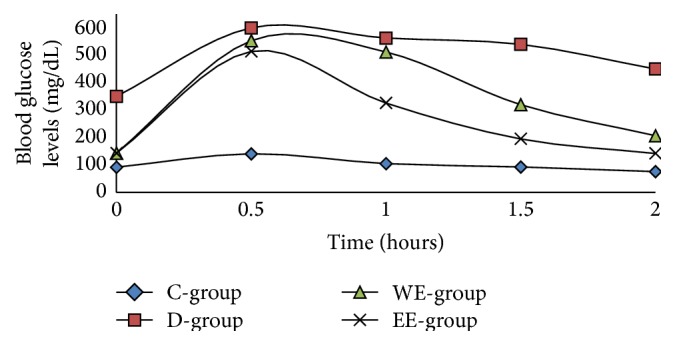
Changes in blood glucose level during oral glucose tolerance test (OGTT).

The time-course changes in the blood glucose levels during the oral glucose tolerance test (0–120 min) of all groups were shown in Figure 2. The blood glucose levels reached the maximum after 30 minutes of administration of 2 g glucose/kg BW and then reduced to initial levels within 2 hours in all groups except diabetic rats that showed severe glucose intolerance throughout the experimental period (0–120 min). However, treatment of diabetic rats with 300 mg/kg BW of either water or ethanolic extracts of* A. maurorum* significantly (*P* ≤ 0.05) lowered their blood glucose levels to the normal level (Figure 2).

Plasma levels of triglycerides (TG), total cholesterol (TC), LDL-cholesterol (LDL-C), and VLDL-cholesterol (VLDL-C) were increased in diabetic rats compared to the control rats (Table 1). On the other hand, HDL-cholesterol (HDL-C) level was decreased in the diabetic rats compared to control group. Oral administration of either water or ethanolic extracts of* A. maurorum* resulted in significant (*P* ≤ 0.05) decreases in the levels of TG, TC, LDL-C, and VLDL-C compared to the diabetic group and increased HDL-C concentration in both WE- and EE-treated diabetic groups compared to nontreated diabetic group (Table 1).

### 3.3. *Alhagi* Extracts and Oxidative Stress

The level of MDA and the activity of GR significantly (*P* ≤ 0.05) increased in hepatic tissues of diabetic rats compared to control group (Table 2). However, the hepatic content of GSH and activities of SOD, GPx, and GST were significantly (*P* ≤ 0.05) decreased in diabetic rats compared to control group (Table 2). Both WE and EE treatments significantly (*P* ≤ 0.05) reduced hepatic MDA level and GR activity compared to the diabetic group. Also, treatments of diabetic rats with either WE or EE improved the antioxidant status of hepatic tissues by increasing GSH level and the activities of SOD, GPx, and GST compared to the diabetic group (Table 2).

### 3.4. Histopathological Studies

#### 3.4.1. Pancreatic Tissues

**Figure 3:**
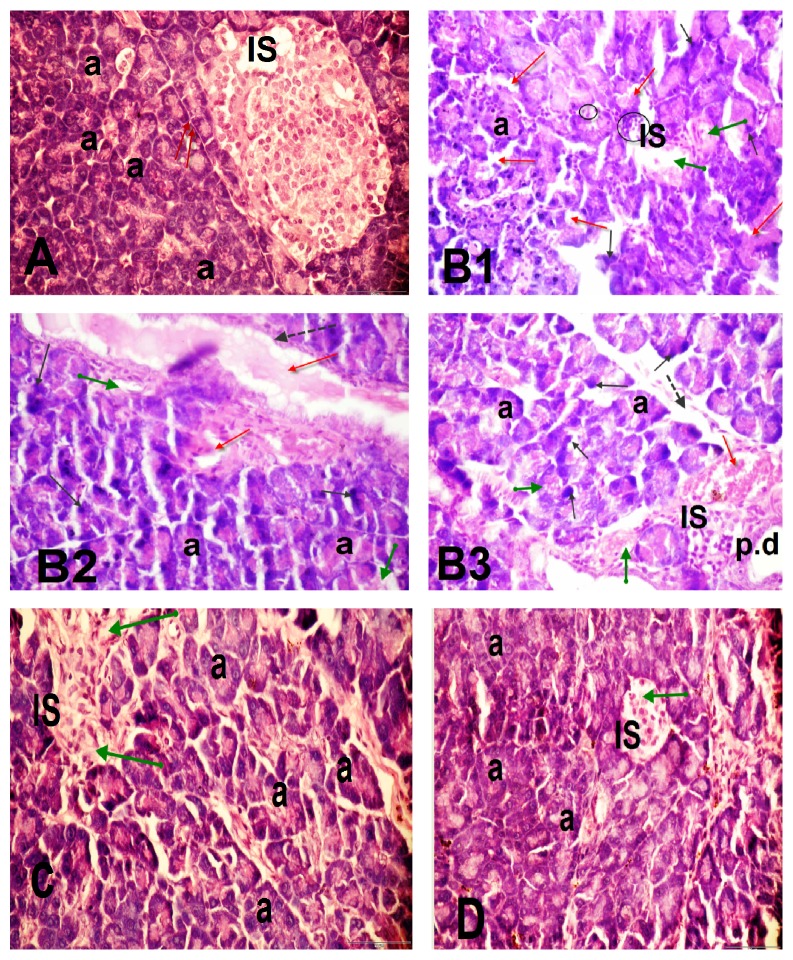
Light micrographs of pancreatic sections of the following. (A) Control rats revealed normal pancreatic architecture; the closely packed pancreatic acini composed of pyramidal shaped cells with rounded nuclei (a), the pale-stained normal islets of Langerhans (IS) scattered in between acini with well-preserved cytoplasm, and nucleus normal interlobular connective tissue septa (red arrow). B1, B2, and B3 represent STZ-diabetic group of rats and showed disturbance of the acinar pattern structure, pyknotic nuclei of some acini cells (black arrow) with severe damage; dilation and thickening of blood vessels (dashed arrow) and congestion of the blood vessels (red arrow) and vacuolated acini (green arrow). Islets with irregular outline, vacuolated cytoplasm (circle), and degeneration of *β*-islet cells (green arrow). C and D represent STZ + WE- and STZ + EE-treated rats and showed a slight reduction in the histological alterations of the pancreatic acini only. H&E, 400x.

Control rats reveal normal pancreatic architecture; the closely packed pancreatic acini were composed of pyramidal shaped cells with rounded nuclei (a), the pale-stained normal islets of Langerhans (IS) scattered between acini with well-preserved cytoplasm, and nucleus normal interlobular connective tissue septa (red arrow), Figure 3(A): Figures 3(B1), 3(B2), and 3(B3) of STZ-diabetic group of rats showing disturbance of the acini pattern structure, pyknotic nuclei of some acini cells (black arrow) with severe damage; dilation, thickening, and congestion of the blood vessels (dotted arrow and red arrow); and vacuolated acini (green arrow). Islets with irregular outline, vacuolated cytoplasm (circle), and degeneration of *β*-islet cells (green arrow) inflammatory cells infiltrate around the pancreatic duct (p.d) (Figures 3(C) and 3(D)): STZ + WE- and STZ + EE-treated rats showing slight histological alterations of the pancreatic acini only.

#### 3.4.2. Hepatic Tissues

**Figure 4:**
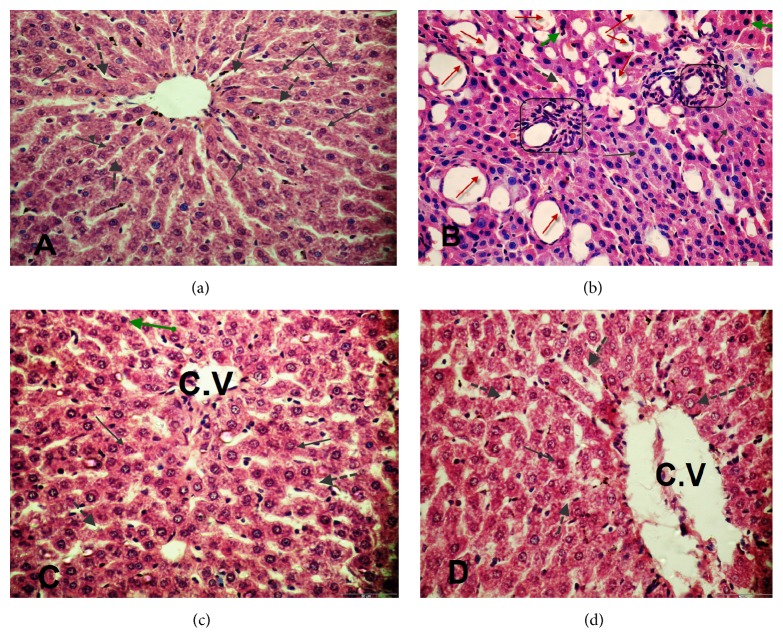
Paraffin sections stained by hematoxylin and eosin for histopathological examination of hepatocytes of rats: (a) liver tissue of control showing normal structure, central vein (C.V), normal arrangement of hepatic cords, normal blood sinusoids (⇢), and hepatocytes (→); (b) liver tissue of diabetic rats (STZ) showing hepatocyte vacuolization and fatty changes (red arrow), necrosis (green arrow), dilation of hepatic sinusoids (⇢) also, bile duct and portal vein (□), and cell infiltration (○); (c and d) liver tissue of diabetic rats + WE and diabetic rats + EE extracts of* Alhagi* showing normal structure, central vein (C.V), normal arrangement of hepatic cords, normal blood sinusoids and hepatocytes, few necroses, less degenerative changes, and vacuolization. H&E, 400x.

The histological examinations of hepatic tissues are represented in Figure 4. The light micrographs of liver tissues demonstrated normal architecture of hepatic cells and central vein and normal blood sinusoids in the control group (Figure 4(a)), while STZ-diabetic rats revealed severe pathological changes including congestion and dilation of hepatic sinusoids. Moreover, portal areas showed hyperplasia in the biliary epithelium and wall thickness of hepatic arteries. Focal aggregations of lymphocytes were also noticed in diabetic rats. The hepatic cells revealed degenerative and necrotic changes. Also, diffuse vacuolar, hydropic degeneration, and hypertrophied Kupffer cells were seen in diabetic rats (Figure 4(b)). However, livers of diabetic rats treated with* A. maurorum* extracts markedly reduced and attenuated the histological changes from severe to moderate alterations (Figures 4(c) and 4(d)).

## 4. Discussion

From ancient times, diabetic patients have used medicinal plants to maintain blood glucose level [30]. In this regard, the present study is extended to show the influence of* A. maurorum* extracts on blood glucose level, oxidative stress, and lipid profile in STZ-induced diabetic rats [31]. Diabetes mellitus type 1 is caused due to lack of insulin secretion [32]. Consistent with this finding, the present study showed a significant reduction in insulin levels in diabetic rats with no recovery after their treatments with either water or ethanolic extracts of* A. maurorum*. The hypoglycemic effects of* A. maurorum* extract were not attributed to regeneration of *β*-cells or to increase of insulin secretion. This finding was confirmed by the histological analysis of pancreatic tissue since diabetic rats showed a reduction in numbers of islets and degeneration of *β*-cells. In agreement with the present study, a selective necrosis of *β*-cells islets of Langerhans of STZ-treated rats has been found [33]. It has been found a variable changes in nuclei of islets of pancreas in diabetic rats and some of them appeared as pyknotic nuclei due to condensation and shrinkage of the nuclear materials [34], and this is in agreement with the finding of the present study. However, treatment of diabetic rats with* A. maurorum* extracts showed a slight attenuation in pancreatic acini only.

It is known that hyperglycemia in both animals and humans with type 1 diabetes results from the increase in hepatic glucose output and the decrease in peripheral glucose utilization [35]. Because* A. maurorum* extracts do not affect *β*-cells regeneration or insulin secretion, the hypoglycemic effects of these extracts may be due to the presence of gallic acid and rutin as major constituents of these extracts. Interestingly, gallic acid increased glucose uptake and enhanced the translocation of GLUT4 at concentrations comparable to the amount of gallic acid. The hypoglycemic effects of* A. maurorum* extracts may be due to presence of phenolic compounds in these extracts. Supporting our finding, it has been found that gallic acid increased glucose uptake via different mechanisms [36]. In addition, it has been reported that quercetin, a flavonoid of* A. maurorum*, increased glucose uptake and increased GLUT4 translocation [37]. In addition, it has been found that rutin was served as a potential agent for glycemic control through enhancement of insulin-dependent receptor kinase activity, thereby inducing the insulin signaling pathway causing increased GLUT4 translocation and increased glucose uptake [38]. In skeletal muscle, rutin significantly increases intracellular calcium concentration which may induce glucose transporter-4 (GLUT-4) translocation with consequent glucose uptake [39].

The liver diseases are more prevalent in the diabetic population [40]. The activities of AST and ALT and the level of bilirubin were increased in diabetic rats. However, treatment of STZ-induced diabetes in rats with either water or ethanolic extract of* A. maurorum* reduced AST and ALT activities and total bilirubin levels compared to diabetic rats which are consistent with the finding of Shaker et al. [41]. The hepatoprotective effect of* A. maurorum* extracts may be due to the presence of flavone structures in the ethanolic extract [42].

Both hypertriglyceridemia and low level of HDL are the most common lipid abnormalities related to diabetes mellitus [43]. In the present study, the diabetic rats exhibited hypertriglyceridemia, hypercholesterolemia, elevated LDL-C, elevated VLDL-C, and reduced HDL-C levels compared to the control group. However, treatment of diabetic rats with either water or ethanolic extracts improved LDL/HDL ratio and lowered TG, TC, and VLDL-C levels. This improvement might be due to the presence of lupeol, a component of* A. maurorum* extract, which plays an important role in normalization of lipid profile [15].

The elevated levels of oxidative stress in diabetic animals are due to autoxidation of glucose, protein glycation, lipid peroxidation, and low activities of antioxidant enzymes [44]. Consistent with this finding, the present study showed that increased MDA level, decreased GSH level, and decreased activities of antioxidant enzymes, such as SOD, GPx, and GST, were seen in livers of the STZ-induced diabetic rats. These results are in agreement with other previous study which showed that glutathione level was decreased in different phases of diabetes [45]. The mechanism of enhancing of oxidative stress might be due to protein glycation and inhibition of antioxidant enzymes activities (superoxide dismutase and glutathione peroxidase) [46]. In addition, it has been found that flaxseed oil diet upregulated expression and induced activities of CAT and SOD and the protein expression of GPx, whereas fish oil diet induced both the activity and expression of CAT in liver of streptozotocin-nicotinamide induced diabetic rats [47]. In the current study,* A. maurorum* extracts alleviated oxidative stress by inducing the activities of antioxidant enzymes (GPx and GST) that were inhibited in diabetic rats. These results are in agreement with other study which reported that the ethanolic extracts of* A. maurorum* ameliorate the oxidative stress by increasing the level of glutathione and decreasing the MDA level [41]. Moreover, the antioxidative effect of* A. maurorum* might be due to the presence of flavonoid compounds [48] such as quercetin, which protects human intestinal cells and hemoglobin against oxidative stress attack [49].

The depletion of GSH level in diabetic rats might be due to its utilization to alleviate the oxidative stress in diabetes [50]. Therefore, the increased activity of GR in the diabetic rats was to compensate the decreased GSH levels through reduction of the oxidized glutathione (GSSG), which might be increased due to the presence of high levels of free radicals in DM. On the other hand, treatment of diabetic rats with* A. maurorum* extracts elevated the GSH levels and restored the activity of glutathione reductase in diabetic rats to its normal levels. The liver is frequently damaged during diabetes, as a consequence of increased levels of oxidative stress and dysregulation of immune function [16]. The degenerative changes in the histology of liver with abnormal localization and infiltration of hepatocytic nuclei were found in STZ-induced diabetes. On the other hand, livers of the diabetic rats that were treated with* A. maurorum* extracts revealed that most of these changes were attenuated from severe to moderate alterations which are in agreement with the finding of Alqasoumi et al. [51].

In conclusion,* A. maurorum* extracts decreased blood glucose levels and increased antioxidant enzymes activities in diabetic rats. In addition,* A. maurorum* extracts suppressed the level of free radicals and dyslipidemia in diabetic rats and consequently could alleviate complications of TD. Further studies to isolate the active components of* A. maurorum* are needed for treatment of diabetes mellitus.

## Figures and Tables

**Table 1:** Changes in levels of liver function markers, blood glucose levels, and lipid profile in plasma of diabetic rats treated with either water or ethanolic extract of *Alhagi maurorum*.

Parameters	Animals treatments			
	Control	STZ-group	STZ-WE-group	STZ-EE-group
AST (U/L)	18.67 ± 1.41^c^	503.46 ± 29.61^a^	24.33 ± 2.62^b^	21.57 ± 1.23^b^
ALT (U/L)	6.141 ± .079^c^	103.63 ± 12.563^a^	9.64 ± 1.479^b^	8.62 ± 1.216^b^
Bilirubin (mg/dL)	0.51 ± 0.03^c^	6.982 ± .18^a^	0.43 ± 0.04^b^	0.72 ± 0.04^b^
Cholesterol (mg/dL)	104.76 ± 7.6^c^	203.14 ± 9.03^a^	63.92 ± 4.76^b^	90.47 ± 7.90^b^
LDL-C (mg/dL)	83.58 ± 7.01^c^	173.18 ± 8.47^a^	54.60 ± 6.41^b^	56.51 ± 4.88^b^
HDL-C (mg/dL)	88.26 ± 8.15^c^	40.246 ± 1.774^a^	73.965 ± 6.927^b^	63.499 ± 7.549^b^
LDL/HDL ratio	1.1 ± 0.14^c^	4.35 ± 0.23^a^	0.76 ± 0.08^b^	0.99 ± 0.13^b^
VLDL-C (mg/dL)	24.37 ± 2.15^c^	97.52 ± 12.51^a^	10.14 ± 1.60^b^	16.70 ± 2.24^b^
Triglyceride (mg/dL)	121.83 ± 10.7^c^	487.60 ± 62.57^a^	50.68 ± 8.02^c^	83.52 ± 11.21^b^
Glucose (mg/dL)	90.81 ± 5.36^c^	437.6 ± 14.10^a^	106.83 ± 8.57^b^	82.24 ± 7.09^b^

Values are expressed as mean ± SE of 10 rats in each group.

^abcd^Mean values within a row not sharing the same superscript letters were significantly different, *P* < 0.05.

**Table 2:** Changes in level of free radicals and activities of antioxidant enzymes in liver of diabetic rats treated with either water or ethanolic extract of *Alhagi maurorum*.

Parameters	Animals treatments			
	Control group	STZ-group	STZ-WE-group	STZ-EE-group
MDA (nmoles/g tissue)	551.20 ± 64.29^a^	1222.40 ± 101.9^d^	845.00 ± 31.32^b^	647.80 ± 52.73^c^
GSH (nmoles/g tissue)	876.35 ± 57.55^d^	424.15 ± 48.34^a^	1103.66 ± 105^b^	1317.82 ± 133.8^c^
GPx (mU/mg protein)	634.12 ± 61.31^d^	355.28 ± 41.68^a^	474.50 ± 25.37^b^	506.19 ± 21.51^c^
GR (mU/mg protein)	15.04 ± 0.53^b^	23.35 ± 1.85^a^	13.62 ± 1.09^b^	15.98 ± 0.96^b^
GST (mU/mg protein)	17.95 ± 1.16^b^	13.81 ± 0.45^a^	17.05 ± 1.67^b^	15.57 ± 1.01^d^
SOD (U/mg protein)	216.57 ± 19.01^b^	139.94 ± 7.39^a^	197.79 ± 32.37^b^	205.50 ± 16.6^b^

Values are expressed as mean ± SE of 10 rats in each group.

^abcd^Mean values within a row not sharing the same superscript letters were significantly different, *P* < 0.05.

## Abbreviations

DM: Diabetes mellitus
SOD: Superoxide dismutase
GPx: Glutathione peroxidase
GR: Glutathione reductase
GST: Glutathione-S-transferase
TG: Triacylglycerol
TC: Total cholesterol
LDL-C: Low density lipoprotein-cholesterol
HDL-C: High density lipoprotein-cholesterol
VLDL-C: Very low density lipoprotein-cholesterol
ROS: Reactive oxygen species
MDA: Malondialdehyde
STZ: Streptozotocin
ALT: Alanine aminotransferase
AST: Aspartate aminotransferase
OGTT: Oral glucose tolerance test.
